# Detection methods for influenza A H1N1 virus with special reference to biosensors: a review

**DOI:** 10.1042/BSR20193852

**Published:** 2020-02-04

**Authors:** Ravina, Anita Dalal, Hari Mohan, Minakshi Prasad, C.S. Pundir

**Affiliations:** 1 Centre for Medical Biotechnology, Maharshi Dayanand University, Rohtak, Haryana 124001, India; 2 DCR University of Science and Technology, Murthal, Sonepat, Haryana 131039, India; 3 Department of Animal Biotechnology, Lala Lajpat Rai University of Veterinary and Animal Sciences, Hisar, Haryana 125004, India; 4 Department of Biochemistry, Maharshi Dayanand University, Rohtak, Haryana 124001, India

**Keywords:** A (H1N1), DNA biosensors, Orthomyxoviridae, Real Time-PCR, RT-LAMP, Swine flu

## Abstract

H1N1 (Swine flu) is caused by influenza A virus, which is a member of *Orthomyxoviridae* family. Transmission of H1N1 occurs from human to human through air or sometimes from pigs to humans. The influenza virus has different RNA segments, which can reassert to make new virus strain with the possibility to create an outbreak in unimmunized people. Gene reassortment is a process through which new strains are emerging in pigs, as it has specific receptors for both human influenza and avian influenza viruses. H1N1 binds specifically with an α-2,6 glycosidic bond, which is present in human respiratory tract cells as well as in pigs. Considering the fact of fast multiplication of viruses inside the living cells, rapid detection methods need an hour. Currently, WHO recommended methods for the detection of swine flu include real-time PCR in specific testing centres that take 3–4 h. More recently, a number of methods such as Antigen–Antibody or RT-LAMP and DNA biosensors have also been developed that are rapid and more sensitive. This review describes the various challenges in the diagnosis of H1N1, and merits and demerits of conventional vis-à-vis latest methods with special emphasis on biosensors.

## Introduction

**Figure 1:**
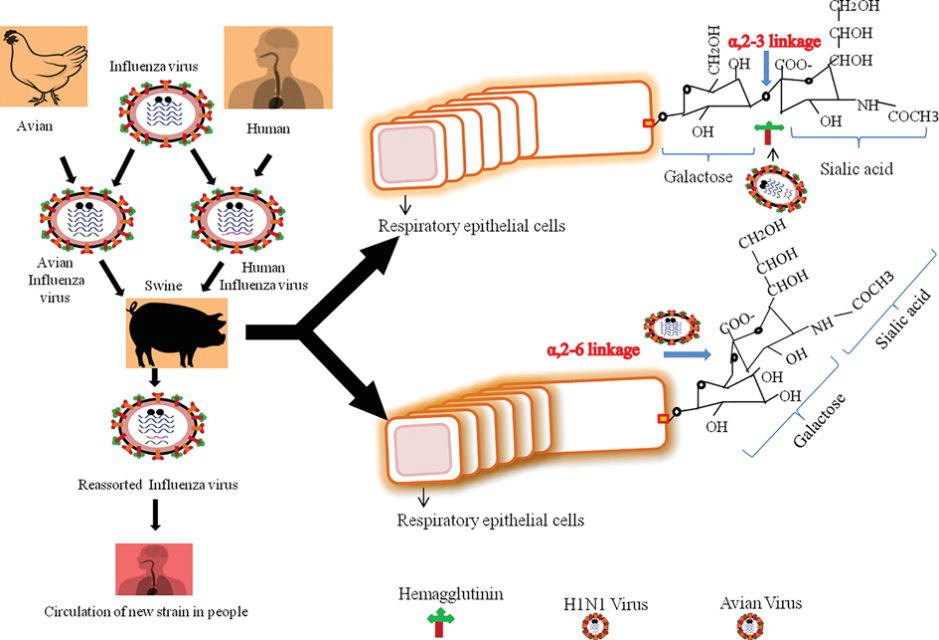
A basic mechanism showing new strain development of H1N1 by antigenic shift, and its transmission in human Pig has receptors for avian influenza virus as well as for human influenza virus (α-2,3 bond between sialic acid and galactose, α-2,6 bond between sialic acid and galactose, respectively).

Virus borne infectious diseases are the major concern of today’s world, every day new viruses are causing outbreaks and old viruses are becoming stronger. One such virus is influenza virus, which has four types A, B, C and D. Importantly, influenza type A and B are responsible for major outbreaks compared with type C and D, which are less virulent, genetically stable and infect animals only. Based on antigenic composition, influenza A is further classified into different subtypes depending on two major surface antigens, hemagglutinin (HA) and neuraminidase (NA), which are further classified into several types. HA is classified into 18 different types and NA is classified into 11 different types [1,2]. Swine flu is a fatal contagious respiratory disease caused by A (H1N1) pdm09 influenza virus of *Orthomyxoviridae* family. In April 2009, a novel Influenza virus (H1N1) emerged in Mexico, which aired all around the world within week and WHO declared it global pandemic of phase 6 level on 11 June 2009, which ended on 10 August 2010 with several deaths worldwide (WHO report). It was first detected in 1930 in pigs as classical swine H1N1 in the United States after 1918 pandemic of H1N1 [3,4]. This virus was a result of quadruple reassortment in triply assorted virus with Eurasian (Europe and Asia) swine virus, in which one of the viruses was descendent of 1918 strain [5]. It is a negative sense single-stranded RNA virus having eight segments, which codes for transcriptase, surface glycoproteins, hemagglutunin (HA), neuraminidase (NA), matrix protein and nucleocapsid proteins [6,7,8]. It is a spherical virus with 80–120 nm filament with symmetric helical nucleocapsid. HA and NA are responsible for the binding of a virus with sialic acid of respiratory tract cells and release of viral progeny from infected cells, respectively. HA of different viruses recognizes different receptors on host cells in which human influenza virus specifically recognizes α, 2-6 glycosidic bond between sialic acid and galactose on respiratory cells but avian influenza virus specifically binds with α, 2-3 glycosidic bond. Pigs have both types of sialic acid receptors for antigens, which are possessed by virus of avian or human susceptibility. Thus, they act as a mixing vessel and provide a site for genetic re-assortment, which results into antigenic shift [9,10,11]. A general diagram is shown in Figure 1.

Symptoms of influenza are similar to that of common influenza virus and include fever above 104°F for more than 3 days, headache, coughing, sore throat, vomiting, chest pain, hypotension, severe dehydration and nausea [12]. Influenza was reported first time with certainty in 1932 but before that it was reported in great historical Greek writings of 412 BC and later on many times in each century. In the 20th century, pandemics occurred four times, first time reported in Spain known as Spanish flu (H1N1) and responsible for deaths of approximately 50–100 million people all around the world [13]. Initially, it was assumed as a disease of pigs but later when pigs and human both were infected at the same time, then it was speculated that the disease transmits from pigs to human [14] when isolated first time in 1931 in a laboratory from infected pig by [4]. After 40 years in 1957, another subtype (H2N2) caused pandemic and was responsible for the death of 1–2 million people. It was first detected in China [15] in February 1957 and within 5 months it was found in 20 countries [16]. It was a mild influenza pandemic with a fatality rate of 0.67% [17]. After a decade, new subtype of influenza A (H3N2) caused pandemic in Hong Kong in 1968 in which 500,000–2 million deaths were reported worldwide [18,19]. After that, in April 2009, a novel influenza virus emerged at pandemic level in Mexico of phase 6 declared by World Health Organization (WHO) [20,21]. Every year Influenza A (H1N1)pdm09 takes thousands of lives worldwide and in 2019, according to national centre for disease control (NCDC) of India, 17,366 suspected cases of swine flu were reported with 530 confirmed deaths till 3 March. These numbers are increasing day by day (NCDC, Seasonal Influenza (H1N1), 2019) [22].

Vaccines are available for influenza virus presenting an effective tool but have to be structured every year to cover all changes due to antigenic drift in RNA. A number of changes have been performed recently to increase effectiveness of immune system of body, and to speed up antibody production in case of a seasonal and pandemic emergence. Importantly, universal influenza virus vaccine development is currently in its preclinical and clinical phase [23]. There are many diagnosis methods like enzyme linked immunosorbent assay (ELISA), complement fixation test (CF), double immunodiffusion (DID), hemahgglutinin inhibition (HI) and real-time polymerase chain reaction (RT-PCR), but some takes 4–5 days for confirmation and some are less specific. The severity of infection increases with time therefore, there is a need for early detection of infection. RT-PCR is a method of choice for confirmation of disease but it is labour intensive and costly. This assay shows 97% accuracy and limit of detection (L.O.D) is 0.1–102 PFU/ml. This method is specific, accurate and highly sensitive for all strains of influenza A, but it also takes time [24–27]. Biosensors hold good capability to convert today’s diagnostic methods into fast analytical powers by reconstituting their sensing behaviours for the detection of any nano-sized objects, like antibody, biomolecules and pathogens. Therefore, current sensing methods need a continuous upgradation to solve all growing challenges for the diagnosis of a virus. A good description was featured to cover up all challenges, the principle and types of biosensors and their applications in the diagnosis of distinct infectious diseases [28]. Currently, nanotechnology based sensors are used for rapid, sensitive and cost-effective diagnostics of pathogens. Different nanomaterials have different applications and promote interactions between these nanomaterials and the virus, which help in developing portable biosensing tool for electro-analytical analysis for effective detection of the influenza virus [29]. In 2018, an immunosensor for the detection of influenza virus H9N2 was developed. Antibodies against matrix protein 2 (M2) were attached to iron magnetic nanoparticles (MNPs) for isolation of virus from allantoic fluid. Then another biomolecule, Fetuin A, was attached to gold nanoparticles (AuNPs), and used for the analysis of the virus using the benefit of fetuin–hemagglutinin interaction. The isolated MNP-Influenza virus-AuNP complex was treated with an acid solution and AuNPs were electrodeposited onto a screen printed carbon electrode. The sensor can tell influenza virus A/H9N2 at < 16 HAU titer, which is proportional to current signal [30]. In this review, we are presenting detailed information about all diagnostic methods, old as well as new for A(H1N1)pdm09 (swine flu) detection with special emphasis on biosensors.

## Diagnostic methods for A (H1N1)

**Figure 2:**
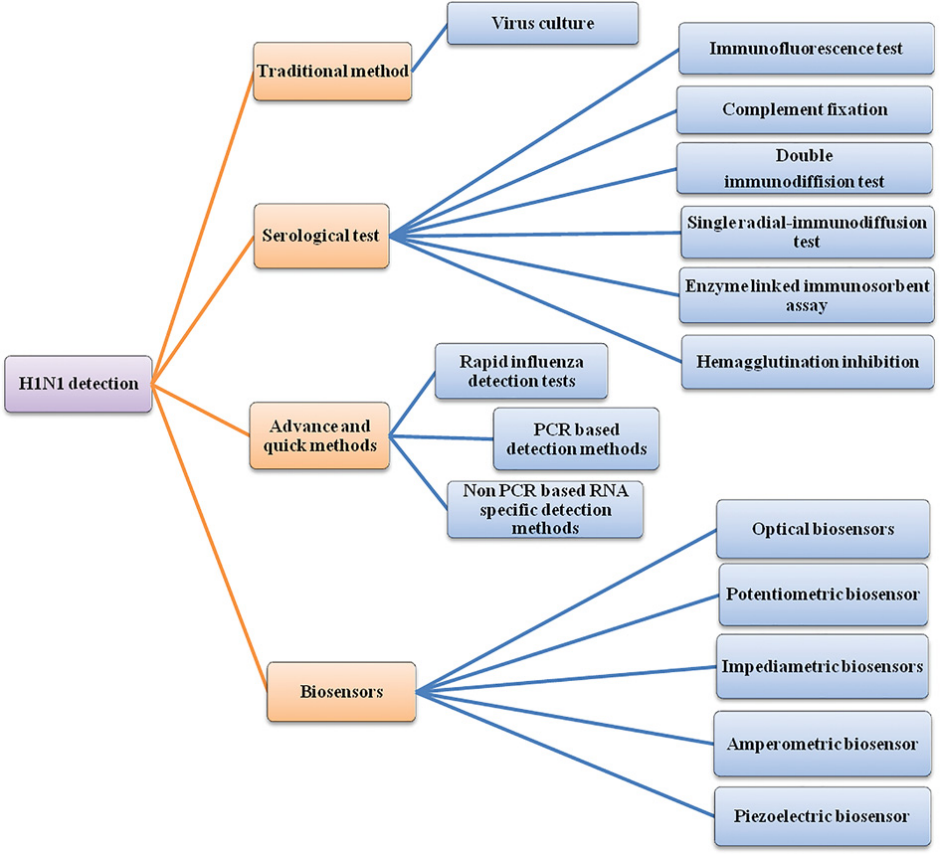
A generalized representation of all different area of detection methods for H1N1

It is challenging to diagnose pathogen in case of influenza, because it shows similar symptoms like rhinoviruses, parainfluenza, adenoviruses and respiratory syncytial virus. Influenza virus spreads quickly within 24 h and can be collected within 2–5 days from nasal and tracheal–throat swabs that are introduced in transport media to keep them alive until transportation to specific laboratories and media sample to detect influenza quickly. A large number of tests are possible for detection, which have different specificity based on the type of detection factor that can be antibody production, direct culturing in cell and nucleic acid amplification detection like RT-PCR and RT-LAMP as shown in Figure 2. These methods help in early treatment of a patient before converting infection into a severe case. All available detection methods for detection of A (H1N1) pdm09 with their analytic characteristics, merits and demerits are mentioned in Tables 1 and 2. Earlier, Chauhan et al. [31] reviewed some methods of influenza A(H1N1) detection such as viral culture, rapid antigen tests, direct immune fluorescence (DFA) and RT-PCR.

**Table 1:** Various kits for H1N1 detection

S.No.	Name of diagnostic method	Target	Sensitivity	Specificity	Reference
1	Immunochromatographic assay based rapid diagnostic kits	NP protein	2 × 10^5^ viral copies/kit	100%	[101]
2	SD Bioline Influenza Ag A/B/A(HINI) Pandemic	–	77%	100%	[102]
3	RapidSTRIPE test	HA	88%	94%	[103]
4	Immunochromatography (IC) rapid diagnostic test kits	HA, NP	49.4%, 79.5%	93%, 100%	[104]
5	Rapid fluorescent immunochromatographic strip test	NP protein	85.29%	100%	[105]

Notes: NP, nucleoprotein; HA, hemagglutinin.

**Table 2:** Various biosensors for detection of A (H1N1) virus

S.N.o	Sensor type	Gene/protein	Sensitivity/L.O.D	Sample type	Detection time	References
1	AuNP immunosensor	HA, NA antibody	50.5 pg/ml	H1N1 virus	–	[54]
2	Fluorescent immunosensor	HA/fusion antibody	Not reported	H1N1 Virus	–	[73]
3	Surface plasmon resonance	HA	4.5 pmol l^-1^	H1N1 Virus	–	[74]
4	Immunosensor /SPR	HA /antibody	30 PFU/ml	labelled anti-HA	20 min	[75]
5	Immunosensor	M1/polyclonal antibody	80–100 virions/µl	H1N1 virus	30 min	[79]
6	PEDOT with galatcose	HA binding	0.12, 0.013 HAU	H1N1 virus	–	[80]
7	BDD	M1 antibody	1 fg/ml	antibody-M1	5 min	[83]
8	SiO_2_-IO	HA antibody	10^3–^10^5^ PFU	H1N1 virus	–	[84]
9	SWCNT immunoassay	anti- HIN1	180 TCID50ml	H1N1 Virus	–	[88]
10	Impedance aptasensor	DNA aptamer	0.9 pg/µl	H1N1 virus	–	[89]
11	DPM-coated gold electrode	His_6_-H1 HA	1 × 10^9^ to 1 × 10^8^ fold	HA antibody in sera	–	[90]
12	QCM immunosensor	Anti-MA	1 × 10^3^ pfu/ml	H1N1 virus	>100 min	[95]
13	FET Biosensor	HA binding	6000 HA mol/20 µl	HA protein	–	[112]

Notes: HAU, hemagglutinin unit; TCID, tissue culture infective dose; PFU, plaque forming unit; PEDOT, Poly (3,4-ethylenedioxythiophene); DPM, dipyrromethene; BDD, Boron doped diamond; HA, hemagglutinin; MA. Matrix.

The detection methods of influenza A(H1N1) can be classified as follow:

1. Clinical diagnosis
  - Virus culture
  - Immunofluorescence test
    - Complement fixation
    - Double immunodiffision test
    - Single radial-immunodiffusion test
    - Enzyme linked immunosorbent assay
    - Hemagglutination inhibition
2. Surveillance method
  - Advance and quick methods
    - PCR-based detection methods
    - Non PCR based RNA specific detection methods
  - Biosensors
    - Optical biosensors
    - Potentiometric biosensor
    - Impediametric biosensors
    - Amperometric biosensor
    - Piezoelectric biosensor
    - Magnetic biosensor
    - Thermometric biosensor

### Clinical diagnosis

A viral infection stimulates the immune system in the body that further results in the production of antibodies, and the level of antibodies is reported to increase between 8th and 14th day of viral infection [32]. Checking the presence and measuring the concentration of these antibodies can be used as a diagnostic tool for influenza virus. These antibodies are specific; however, the immune system takes time to produce these antibodies at a detectable level thus reduces the chances of decreases. There are several tests are based on serological analysis, complement fixation, double immunodiffusion, hemagglutination inhibition assay (HIA) test and enzyme immunoassay test (EIA). Some biosensors are also reported, which are based on same antigen–antibody binding affinity.

#### Virus culture

Culturing of the virus in cells shows changes in cells morphology, behaviour etc. The major cell types that have been used are Madin Darby canine kidney (MDCK), monkey kidney cells and A549 cells. Virus samples are used to infect the established cell lines for 7–10 days to see the cytopathic effect on cells and different cells show different levels of effect with 100% specificity and 86–94% sensitivity [33]. New commercial mixed cell lines (R-Mix cells, R-mix Too) are also available, which have more sensitivity than other cell lines and take less time. These are hybrid of A549 with MDCK cells and Mink Lung epithelial cells, respectively (microlab). R-mix Too cells were used for the detection of A(H1N1)pdm09 using kit D3 Ultra 2009 H1N1 ID kit [34]. Confirmation has been done by typing of virus with fluorescent antibodies, which could be easily seen by immunofluorescence microscopy. It was the only method before the development of other methods [35]. However, many labs also used this method with other methods in parallel, as it require many days in the detection of pathogen type, so cannot be used for timely bound detection.

#### Immunofluorescence tests

Immunofluorescence method takes 2–4 h in detecting influenza virus; cells from the sample are fixed on a glass slides, stained and bioconjugated with antibodies with the fluorescent dye and it takes 2–4 h for detection in microscope. Sensitivity and specificity is 70–100% and 80–100%, respectively [36]. Polyclonal or monoclonal antibody produced in an animal against whole virus inoculation can be used for the detection of the virus. A tissue sample infected with viruses are chilled before processing, and the procurement of the results of this method takes several hours [37]. So, the maintenance of these tissues also becomes problematic. These methods are less expensive and provide fast response but the sensitivity is not as high as that of PCR. Also, there is a chance of infection to the laboratory personnel.

#### Complement fixation

It was a traditional method for influenza virus detection using anti-sera for two stable, type specific antigens (nucleoprotein and matrix protein). These antigens are same in all strains of same type of virus. Nucleoprotein and matrix protein are the most stable antigens that have been used for the typing of virus [38]. This test detects the presence of complement fixing antibodies in patient serum. The concentration titer of complement fixing antibodies increases only when infection happens with influenza virus but remains constant or change negligibly with other upper respiratory virus infections [39,40]. Complement fixation test has two steps: (1) complement fixation stage and (2) indicator stage. The first step involves the addition of antigen and complement in an inactivated serum for a defined period. Serum is pre-heated at 56°C for one-half hour to inactivate any anti-complementary molecules. The second step involves the addition of erythrocytes (sheep cells) to check the hemolysis. If antibodies are absent from sera complement remains free and hemolysis of erythrocytes takes place [41]. Different dilutions of antigen or antibodies are used for the detection of influenza [42]. This test is useful in detailing the group specificity and also a number of pathogens can be checked at a single time. However, it is time consuming, laborious and requires specific and costly reagents. Another limitation of this test is that it cannot distinguish different strain of the same type of virus [43,44].

#### Double immunodiffusion test

Double immunodiffusion (DI) for typing of influenza viruses was performed using 1–1.5% agarose [45]. Separate wells were used for typing of influenza A and B in single time using nucleoprotein and matrix protein. Reference antigens and antiserums were diffused in selected wells. Strains that were to be typed were diffused in separate wells for both type A and type B for successful identification of all strains. DID can type virus quite sensitively but virus have to be cultured before typing and low virus titer also limit its use commercially. It is also time consuming and laborious method.

#### Single radial-immunodiffusion test

This method can be used to check the antigenicity of virus hemagglutinin and content of hemagglutinin in test antigens [46,47] and also provides extra control for antigenic structure of vaccine [48]. It is a simple process, in which specific antibodies are separated from serum against HA and antigen is poured in wells of agarose gel. Diffusion of antigens in gel is responsible for the formation of an annulus of precipitation of antigen–antibody. A series of different known concentration of antigen in agarose helps in determining the concentration of unknown antigen or virus titre. It can detect antigenic differences in HA that cannot be detected by DI. It is reproducible, simple and sensitive for antigenic composition and used for antigeniclly active antigen detection [49].

#### Enzyme linked immunosorbent assay (ELISA)

ELISA is a very specific and sensitive method for influenza detection. In 1993, a sandwich ELISA was tested in which monoclonal anti-NP antibody attached on plate wells coupled with biotinylated polyclonal anti- A(H1N1)pdm09 whole virus antibodies developed in rabbit was used for detection of nucleoprotein antigen of influenza A virus. This method could detect 10 ng/ml of pure culture. Sensitivity was high because of the use of biotin-avidin signal amplification method [50]. A new sandwich ELISA based diagnostic study was performed during 2009 A(H1N1)pdm09 influenza pandemic. In this ELISA, specific monoclonal antibodies and horseradish-peroxidase linked rabbit anti-HA polyclonal antibodies against HA protein were used. This kit was developed by Xiamen University, China. They reported overall sensitivity (0.57) was higher of ELISA than rapid influenza diagnostic kit QuickVue Influenza A+B test (0.43). The sensitivity of both test varied according to the different viral load. It was 100% at high viral load and then decreased with low viral load. ELISA sensitivity decreases after 10^5^ (log 10/ml) viral load but QuickVue Influenza A+B test sensitivity decreases after 10^7^ (log 10/ml) viral load [51]. In a different study, 1086 sera were analysed from 43 swine herds for different reference strains (H1N1, H3N2, H1N2, H1N1pdm) using ELISA with HI test and it was found that ELISA sensitivity and specificity were higher than HI that were 72.65% and 63.01%, respectively, for the detection of these strains [52,53]. According to these studies, ELISA cannot detect infection at an early stage. A new ELISA-based immunosensor was developed using AuNP (gold nanoparticles) for improved sensitivity. EDC-NHS was used for the binding of anti-HA with AuNP that was already treated with a layer of formic acid (HCOOH). A(H1N1)pdm09 viruses having HA on surface binds with antibodies and secondary antibodies conjugated with (+) AuNP against NA antigen were used. Electrostatic interactions between nanoparticles and antibodies help in increasing the surface area for antibodies that helps in increasing sensitivity. More the number of antibodies binds with nanoparticles, and more antigens can be captured that help in increasing the sensitivity of the procedure. The catalytic activity of gold nanoparticles toward TMB-H_2_O_2_ makes it oxidized, which further causes a change in colour and gives a signal [54].

#### Hemagglutination inhibition

Hemagglutinin inhibition is an assay that was used to check the virus antigenic type, subtype classification specificity of antibodies for hemagglutinin subtypes and to confirm the infection of influenza [55,56]. It is a simple and easy method for detection that requires simple technique and material. Hemagglutination is a property of hemagglutinin antigen found on the surface of virus and can bind with sialic acid of RBCs to form a complex that is known as hemagglutination reaction [57]. In this assay, hemagglutination is inhibited by adding antibodies specific for hemagglutinin that prevents antigen to bind with RBC and to form complex. Different dilutions of antibody were prepared and poured into well which already contain antigens. A positive result does not show agglutination in well. Reproducibility of HI is very low and sometimes cross reaction with other viruses also occurs. Standardization of HI assay can increase reproducibility of this test [58]. In a study, 120 people were tested for current strain of influenza using HI and CF and concluded that HI is more sensitive but less specific than CF [59]. In another study, HI is suggested to be better for subtype detection of influenza than EIA and CF [60]. HI provides better results than CF in detecting the response of antibodies for influenza vaccines in which HI shows 91.2% sensitivity, 25.7% specificity as compared which 38.7% sensitivity and 85% specificity of CF [61].

### Advance and quick methods for influenza detection

#### Rapid influenza detection tests (RIDTs)

RIDT is also known as point of care immunoassay based detection test for influenza virus that can detect virus within 30 min and helps in timely treatment of patient. It detects viral antigens mostly nucleoprotein in respiratory samples and gives coloured signal. RIDTs can be tested in three formats- dipstick, cassettes and cards. These tests are helpful in fast detection but can produce false positive results with poor sensitivity. Analytical sensitivity differs according to the specimen type, skills of technicians, time after infection and age of patient etc. In a study, NanoSign Influenza A/B kit was used for testing of 1023 samples and the resulted data were then compared with conventional RT-PCR. It was observed that the sensitivity and specificity of both the tests were found 79.4% and 97.2% respectively with 94% concordance [62]. Another study reported the results of QuickVue Influenza A+B(Quidel) rapid influenza antigen test that was performed on 1538 patients and the sensitivity and specificity was found 20% and 99%, respectively [63]. Although RIDT can easily differentiate between influenza A and influenza B; however, it cannot differentiate between subtypes of influenza A that makes it less favourable and further testing is required for confirmation [64]. In an another study thar performed during 2009 influenza A H1N1 outbreak, a comparison was carried out between TruFlu rapid influenza A and B, rapid assay Directigen EZ detection and Direct Immuno Flourecence assay (DFA) test with Real-time PCR for detection of swine origin influenza virus (S-OIV). Sensitivity of both rapid influenza test and DFA test were 9.7%, 20.6%, 32.35% respectively and the specificity were 98.2%, 99%, 99% respectively in comparison with RT-PCR. According to all these studies, these POC test are less sensitive and cannot be used for 100% accuracy [65].

#### PCR based detection methods

Polymerase chain reaction (PCR) is the method of choice that is currently in use for swine flu detection and for many other pathogens also. It can be reverse transcriptase based PCR and real-time PCR. Every time WHO publish guidelines for the detection of swine flu. Basically in a two step RT-PCR, RNA first has to be converted into cDNA using reverse transcriptase enzymes and then in second step random hexamers are used for amplification of segments. Whereas, in one step RT-PCR gene specific primers with reverse transcriptase enzyme were added in one flow. Virus type specific differentiation is carried out using matrix gene amplification because matrix gene shows conservation in virus that makes it useful for the typing of influenza. Subtyping is carried out with HA gene using specific primers. These methods are very sensitive, but take long time for confirmation. Testing is carried out at Specific centres which further delay diagnosis and make condition of patient severe. In 1999, a reverse transcription based polymerase chain reaction was developed to differentiate between two different subtypes of influenza A H1 and H3. Test was held in both for egg derived isolates and also from lung tissue homogenates. Positive samples were confirmed using immunological assays and it was found that sensitivity was 88.2%, 70% and specificity was 100%, 95.2% in case of egg fluids and lung tissue homogenates respectively [66]. A new real-time PCR and conventional RT-PCR assay was developed for detection of novel A(H1N1)pdm09, which can discriminate seasonal H1N1 viruses from other subtypes and also between other swine viruses and human H1 types successfully [67]. A number of studies also reported development of multiplex real-time PCR assay based on matrix gene for detection of reverse zoonotic influenza infection H1N1 pdm2009 from endemic swine influenza viruses [68]. These PCR assays provide good results but on the other hand they are laborious and expensive too.

#### Non PCR-based RNA specific detection methods

Loop-mediated isothermal amplification is an excellent, specific and sensitive one step amplification method for detection of pathogens. Its fundamental property is that it does not require any high profile machine for processing like real-time PCR and conventional PCR for amplification. It can amplify even in hot water bath. By adding reverse transcriptase, it can also be applied for the detection of RNA viruses. Generally, four primers were used that recognizes six different sites on target DNA [69]. A specific RT-LAMP method was developed for specific evaluation and subtyping of influenza viruses by [70]. In this method, two sets of forward primer and two sets of inner primers were used in which one set recognizes outer region and the other set recognizes inner region and gives a result with 93.8% specificity. Some researchers also add two extra primers for loop regions that increase its sensitivity and specificity. All primers, DNA polymerase (isolated from *Bacillus stearothermophilus*), reverse transcriptase (avian myeloblastosis virus (AMV)), template RNA with all buffers are included in a single reaction tube like PCR, but it follows a different amplifying process. Further, strand displacement is considered to be the most important step as it determines the time for completion of the process.

### Biosensors for H1N1 detection

**Figure 3:**
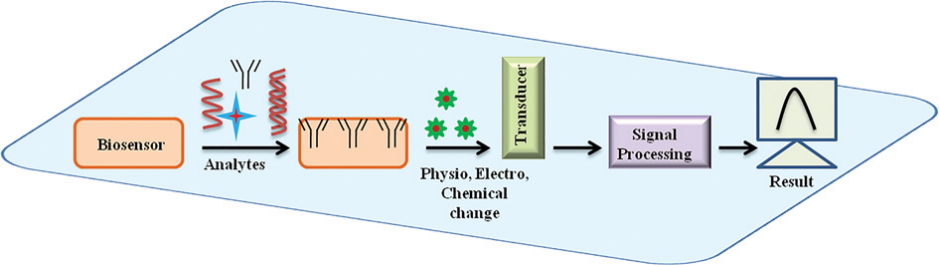
A basic concept of geno and immunosensor

**Figure 4:**
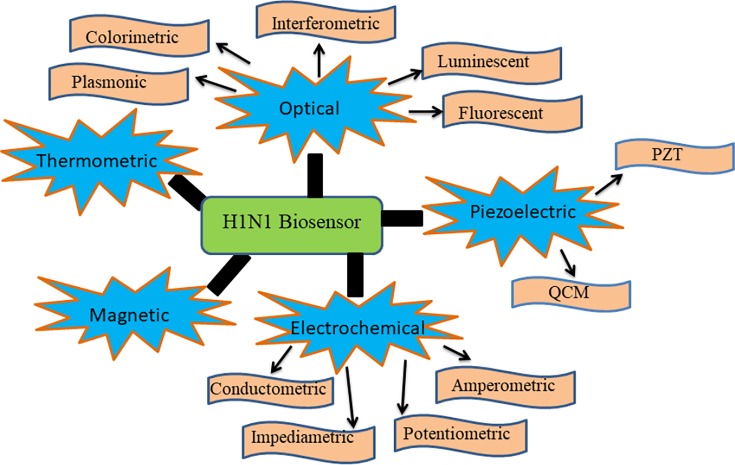
Areas classified for different biosensors development for diagnosis of H1N1

Early detection of dreadful diseases is always necessary, but present methods are not so fast and differentiating; therefore the development of a new method is required that can produce specific as well as quick results. For the first time, biosensor was developed by Clark and Lyon in 1962 for glucose detection in blood using immobilization of glucose oxidase enzyme [71]. Different types of biosensors have been developed to date based on different biological analytes such as antigen–antibody, enzyme based, protein based, DNA–RNA based, thermal and piezoelectric biosensors [72]. The biosensor can sense any biological object like DNA, RNA, PNA, protein, enzyme, antigen and antibody based on their affinity to bind with their complementary segments. A basic biosensor has four components, as shown in Figure 3, a biological analyte, a transducer, an amplifier and a processor. When a biological component binds with a receptor that is already pre-attached to the surface of electrode, it can be enzyme–ligand, antigen–antibody, DNA, RNA that generates a signal in the form of current/charge, heat and gas that is then transformed by a transducer into a readable form. Transducers convert molecular changes in the detectable form and a large number of materials have been used for development of transducers that can be applied for biosensor development. Many nano-sized transducers are available at commercial level that can be carbon nanotubes, gold nanoparticles, semiconductor nanoparticles, perfluorocarbon, silver, organic polymers (MPA, PEI), amino acids, nanofibres and nanotubes. These transducers can provide high sensitivity, high binding affinities and good conductivity and better electrical connections. Biosensors can be classified into different types based on their method of result interpretation, as shown in Figure 4.

There are optical biosensors, electrochemical, piezoelectric and conductometric biosensor. These biosensors can have different variations in term of visual detection methods such as fluorescent biosensor, luminescent, colorimetric and interferometric as shown in Figure 4 in case of optical biosensor. These biosensors can detect changes at microscopic level and a number of studies are reported that involves the detection of pathogens using biosensors. They provide better results in short time with greater sensitivity and high specificity and even at small cost with easy processing in comparison with other conventional methods. Some of the reported biosensors for swine flu detection are as follow-

#### Optical biosensors

Optical biosensors are based on the detection of the pathogen through the change in color or production of color during reaction between analyte and target and a number of variations based on the type of visual method are reported in optical biosensors such as fluorescent biosensor, luminescent, colorimetric and interferometric biosensors. Perceptible changes in optical biosensors occur by two means: change in visual characteristics of sensing area when analyte binds with target as happens in surface plasmon resonance (SPR) or by labelling the analyte with a specific fluorescent molecule that gives visual signals. A fluorescent immunosensor was specially designed for detection of antigen or antibody in sera against A (H1N1) pdm09 [73]. In this study, the plastic based microfluidic sensor was developed that further coated with gold in detection channels of the sensor. They used gold-binding polypeptide (GBP) recombinant influenza hemagglutinin antigen fusion protein as a receptor on surface of the microfluidic chip that has three detection zones for different concentrations. GBP has an excellent binding affinity for gold and does not require any modification on surface of sensor. An antibody with specific fluorescence label (Cy3-labeled anti-H1 Ab) was used as a signal for detection through microchannels. With increasing concentration of fluorescent labeled antibody, the signal also increases, which was verified using confocal microscopy. These immunosensor provide us cost effective, easy handling and it takes less time as compared with other detection methods. Surface plasmon resonance is an optical electronic process where a polarized light hits on the metal surface or at the contact between solutions of different refractive indexes. It is a label free technique that gives information in real time about not only binding capability between protein–protein, DNA, antigen–antibody but also binding kinetics of different molecules at their molecular level. It can give information about the real-time binding of molecules at what rate by interpreting the binding curve. It was proved by using neomembranes made of bovine brain lipid having sialoglycolipids on HPA sensor chip. The binding was best at 30–35°C temperature as compared with 10°C and proved that rate of binding increase with increasing concentration of free NANA (N-acetylneuraminic acid) not only in the presence of only viruses with increased free energy change 3 kJ mol^-1^ during transition complex formation. Thus, SPR is a technique to analyse or measuring binding reaction of molecules at real time [74]. However, the sensitivity of the SPR technique is not good at ultralow concentrations therefore better sensitivity was achieved by using paired surface plasma waves biosensor (PSPWB) with heterodyne technique and integrated with normalization of amplitude. However, it was difficult to avoid drifts and noise which makes its purpose for ultra-low concentration detection difficult. A new improved SPR biosensor with electro-optic modulator (EOM) was used to produce optical heterodyne signals. It was sensor that detects cultured isolated virus in mimic solution with the help of antibody against heamagglurinin (H1). It was a dual channel paired surface plasma waves biosensor that has detection limit 30PFU/ml, calculated by using fitting curve of SPR signals with respect to S-OIV(swine origin influenza A virus) sample concentration. They reported it as a more sensitive method that takes less than 20 min as compared with available rapid influenza diagnostic kit test however, not as sensitive as real-time polymerase chain reaction that have theoretical LOD 3.5 PFU/ml as reported in their study and 1.8 × 102 PFU/ml. It was a good attempt for better sensitivity and early detection [75]. A rapid aptamer based sensor was developed for detection of avian influenza virus (AIV) H5N1 in animals and humans. Aptamer was used as the specific recognition element in a portable surface plasmon resonance (SPR) biosensor in poultry swab samples. The immobilized aptamers captured AIV H5N1 in a sample solution, responsible for an increase in the refraction index (RI). This sensor can detect 0.128–12.8 HAU in 1.5 h of H5N1 [76]. Another study reported a fluorescent aptasensor for the detection of recombinant hemagglutinin (rHA) protein of the H5N1 influenza virus in human serum. Guanine-richen anti-rHA aptamers were captured on the surface of the Ag@SiO_2_ nanoparticles via SELEX technique to make a metal-enhanced fluorescence (MEF) sensor. A fluorescent tag Thiazole orange (TO) was used as to the G-quadruplex secondary structural induced by aptamer-rHA binding event. When rHA protein was absent, TO remains free in the solution with almost no fluorescence emission. When rHA protein was added to the solution, the aptamer strand bound rHA protein to form a stable G-quadruplex complex, which can bind TO and excite the fluorescence emission of TO. Ag@SiO2 nanoparticles enhance the surface plasmon resonance that can be transformed into more efficient fluorescence emission signals, thus amplify the fluorescence signal and can detect a limit of 2 and 3.5 ng/ml in 30 min, without the requirement of fluorophores. [77]. A SPR based biosensor was developed by immobilizing nine respiratory virus-specific oligonucleotides in an SPR chip. To increase the biosensor sensitivity, biotin was used to label the PCR primer and further amplify the signal by introducing streptavidin after hybridization. This biosensor shows the potential to identify these respiratory viruses including influenza A and influenza B, adenovirus, H1N1, parainfluenza virus 1–3 (PIV1, 2, 3), respiratory syncytial virus (RSV) and severe acute respiratory syndrome coronavirus (SARS) from throat swabs [78]. New matrix protein (M1) antibody based gold immunosensor was developed using cyclic voltametry and impedance spectroscopy that can detect whole virus. M1 gene was expressed in *E. coli* and polyclonal antibody was produced in mice and was used for detection A (H1N1) pdm09. They claimed it as a broad spectrum test for all serotypes of influenza A virus with a sensitivity of 80–100 virions/µl and in 30min [79].

#### Potentiometric biosensor

Potentiometric biosensors measures potential difference when a specific current value was applied to the electrochemical cell with the help of potentiostat. In 2017, a study developed a label free A (H1N1) pdm09 detection way based on the concept of HA (on virus surface) binding specificity for human sialic acid. A(H1N1) pdm09 binds with 2, 6 linkage to the galactose residue of sialic acid but avian influenza virus binds with galactose through 2, 3 linkage (2, 3-sialyllactose) of sialic acid. Using this differentiation concept, they developed a new detection method by using a conducting polymer i.e. PEDOT [poly (3, 4-ethylenedioxythiophene)] that is covered and linked with Sia-α2, 6′-Gal-Glu (2,6-sialyllactose) as a recognition site on a surface. Quartz crystal microbalance (QCM) and potentiometry were used for the analysis of virus binding with conducting polymer galactose residue with the sensitivity of two times more magnitude when compared with commercial kits as described by Hai and co-workers [80].

#### Impediametric biosensor

Impedance is also an effective way for analysing the complementary binding of DNA as it provides sensitive results and emerged as a good tool for specific detection of pathogens. It measures resistance produced due to binding of biological element on the surface of electrode with target molecule in sample with respect to potential applied through potentiostat. Several studies for different pathogens are already reported using carbon nanotubes, multiwall carbon nanotube and gold electrode with different surface modifications. According to the study, a comparison of impedance spectra was performed on direct binding of biotinylated target DNA sequence with NH_2_-linked probe and a sandwich scheme in which further attachment with other molecules was performed to enhance the signal. Along with this, they also used streptavidin–gold nanoparticle to increase the impedance signal and sensitivity. The best limit of detection was reported to be 7.5 fmol in sandwich scheme [81]. In a different study, reduced graphene oxide (RGO) based electrochemical immunosensor was developed using EDC-NHS coupling chemistry between COOH group of graphene attached on gold surface of working electrode and NH_2_ of antibody specific for H1 of H1N1 influenza A. Photolithographic technique was used to fabricate glass surface with gold as a working and counter electrode and platinum as a reference electrode to make three electrode system. Then, RGO was used to assemble on working electrode and virus specific antibodies were utilized for electrochemical spectra using different virus concentration for direct detection of whole virus through specific antibodies and reported L.O.D is 0.5 PFU ml^−1^ [82]. In 2017, boron doped diamond (BDD) sensor of three electrode system was developed, in which diazonium salt of 4-aminobenzoic acid was used for creating binding site for attachment of anti-M1 on the surface of electrode. M1 protein was extracted from viruses and used for testing with sensor using K_3_Fe (CN)_6_ in electrochemical impedance spectra reported L.O.D was 1 fg/ml in 5 min response time [83]. In 2018, SiO_2_ - inverse opal (SiO_2_-IO) based biosensor was prepared that showed high sensitivity of 10^3^–10^5^ plaque forming unit (PFU), αHA specific antibodies was immobilized on the surface of SiO_2_-IO by using 3-aminopropyl trimethoxysilane (APTMS) that exposes amine group, and a NHS-PEG_4_-maleimide linker was used for binding of APTMS with thiolated protein G (Cys-ProG). HA specific antibodies were attached with the same procedure with protein G. This biosensor is specific and sensitive but virus binding and analysis takes more than 2 h that makes it less preferable [84]. Thus, a number of studies are still going on based on impediametric study and to achieve good sensitivity and specificity based on different modifications of surface of electrodes.

#### Amperometric sensors

**Figure 5:**
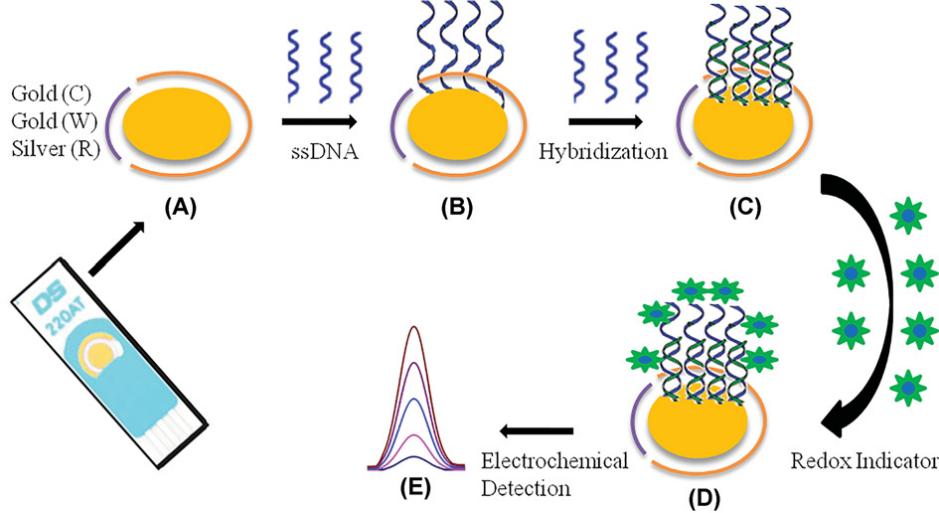
Schematic representation for nucleic acid RNA/DNA based Biosensor development using Au coated three electrode system based on other reported biosensors ssDNA: single-stranded DNA, Au: gold

Amperometric sensors are the sensors that generate response in the form of current when specific potential was applied using potentiostat. Screen printed electrodes are the mainly used electrodes for biosensor development and these can be of two electrode sensor or three electrode sensor. In three electrode system one is working electrode, reference electrode and counter/ Auxillary electrode printed on the surface of metal, glass, paper etc. Potential is applied between working electrode and reference electrode and current is measured between working and counter electrode. Three-electrode based biosensor is more effective because it can manage efficiently potential range for higher current and also provide more area for reaction between analyte and receptor. Reference electrode is used for comparison between applied potential with that of generated potential. Potential gradient of reference electrode is always remains constant. Potential is applied to the working and reference electrode, current is measured between working and reference electrode [85]. Working electrode surface of a sensor is available in printed metal (Au or Pt) or coated with carbon-nanotube. When a segment of nucleic acid (probe), polypeptide, protein and At-Ab is attached to surface of electrode, complementary binding of analyte with pathogen DNA, RNA, PNA (protein nucleic acid) and protein (antibody) takes place. Redox indicators produce current at a certain potential difference between the electrodes after hybridization of probe with sample material. This change in current intensity can be analysed in the form of differential pulse voltametry and cyclic voltametry and in many other forms also with the help of potentiostat. A three-electrode screen printed system using Ag/AgCl as a reference electrode, Pt or gold as an auxiliary electrode and Au electrode as a working electrode has been used for the detection of a number of diseases [86,87]. These methods are easy to manipulate, cost effective, time saving and give accurate results with great sensitivity. Single-wall carbon nanotube (SWCNT) immunoassay biosensor was developed for A (H1N1) pdm09 detection. When surface adsorption of different macromolecules (poly-L-lysine, antibodies, H1N1 virus) occur on surface of immunochips, resistance will increase with concentration of virus and resistance was measured after neglecting the resistance from bare immunochip. A sensitive sensor was developed that have detection sensitivity as 180TCID50/ml after binding of virus with antibody. But it shows false negative result may due to non specific binding with higher concentration feline calicivirus (FCV) [88]. DNA biosensor is an evolving technique in Screen Printed biosensors. Its principle is based on complementary binding of probe (single-stranded DNA segment) with pathogen DNA or RNA that tells the specificity and type of pathogen. A Probe (ssDNA) will be prepared to attach on working electrode. After hybridization of probe with sample genetic material, at a certain potential difference current will generate between the electrodes that cause a change in value of that current that was present before the hybridization. This change in current intensity will give you a signal about the binding type of pathogen. A generalisation of DNA biosensor is shown in Figure 5. Aptamers are a segment of DNA/RNA in its tertiary structure, which can bind specifically with ssDNA, ssRNA, proteins, toxins, carbohydrates and with live cells (pathogens) also. Different shapes and forms (helical structure, loops) are found in aptamers that helps them in fitting or binding specifically with target molecule. Aptamer based gold impediametric biosensor was developed by [89]. These aptamers designed specifically for multivalent binding with inactivated H1N1 viruses and a sensitivity of 0.9 pg/µl was achieved. Another study reported an antibody based DPM-Cu (II) redox (a thiol derivative dipyrromethene) modified with immobilized His_6_-H1 HA on gold surface. It can detect anti-H1 antibodies in mice sera that are diluted from 10^−9^ to 10^−8^ fold specifically and provide better results than ELISA. Osteryoung square wave voltametry was used to track the changes occurred on the surface of electrode with different dilution of antibodies. This voltametry can quantify the difference between reduction and oxidation peak current [90]. A new area of research is PNA-based probe, PNA is a synthetic peptide nucleic acid and can be used as a probe to bind with RNA and DNA through complementary binding. It provides specificity of complimentary binding capability of nucleotides and has greater shelf life than DNA. Many PNA based biosensors has been used in many areas [91]. So, PNA-based biosensors can be a good area for A (H1N1) pdm09 biosensor development.

#### Piezoelectric biosensor

Piezoelectric biosensors showed a change in voltage, when mechanical stress or oscillation is applied to the surface of an electrode that consists of a piezoelectric material. These biosensors based on the physical property of materials and do not have any central symmetry in their molecular structure. Every biosensor that is made up of piezoelectric crystal shows a definite mechanical oscillation after introduction of voltage and a change in oscillation frequency. When an analyte binds specifically (antigen–antibody, DNA–DNA, RNA–DNA, ligand–receptor) with its target that produces a change in oscillation frequency in oscillation circuit and this can be measured as a signal to find out the type of pathogen [92,93]. Mainly anisotropic crystals like aluminium phosphate (berlinite), aluminium nitride, zinc oxide, crystallized topaz (Al_2_SiO_4_(F,OH)_2_), barium and lead titanate, gallium orthophosphate, quartz (SiO_2_), tartrate tetrahydrate (Rochelle salt), polyvinylidene fluoride, poly lactic acids show this type of behaviour because they do not possess any central symmetry. The alternating voltage causes mechanical oscillations of crystal and frequency of oscillations is measured as the crystal is put into oscillation circuit. Analyte or any other mass bound on surface of crystal or more precisely on the surface of electrodes located on the crystal results in change of oscillation frequency. A simple concentration that can change mass on surface after binding on electrode is enough for detection purpose but a slight change in viscosity of medium, density of buffer solutions produce hindrance in actual result. These sensors can be further classified based on the material used for their formation. A study was performed using Quartz crystal microbalance immunosensor in which antibodies (anti-MA) were immobilized onto gold electrode of crystal surface pre-coating the surface with protein A. A change in reflectance frequency shift 30Hz (±5) was measured after 1 h of viral sample introduction on electrode surface but sensitivity increased after using gold nanoparticle conjugated anti-HA and reflectance frequency shift increases 5.8-fold that observed at 102(±11) Hz. When QCM was compared with other detection methods on 67 nasal samples, specificity was 100% and sensitivity is 81% as reported by [94,95]. Another sensor was reported in 2019, by using first radial mode of oscillations of lead zirconate titanate (PZT) piezoelectric discs with a dimension of 2 mm radius and 100 µm thickness covered with a piezoelectric membrane. Synthetic sialylglycopolymer was used for discs modification with a receptor layer, and inserted in a moving virus suspension. Results was analysed by monitoring resonance frequency shift of the disc radial mode with a detection limit of concentrations below 105 virus particles per millilitre. This study reported that Piezo transducers with sialylglycopolymer sensor layers have a long lifetime, a high sensitivity and the possibility of reproducible results [96].

#### Magnetic biosensor

Magnetic biosensors are made up of paramagnetic or super-paramagnetic particles, or crystals. It detects biological interactions by calculating the changes in magnetic properties or magnetically induced effects such as changes in coil inductance, resistance or magneto-optical properties. In 2011, a sensor was developed by using nitrocellulose membrane and magnetic sensors for detection of two influenza A viruses. This combination provides a rapid assay without stop reaction step and does not produce any color as required by other immunochemical methods. Quantitative virus detection was done via magnetic beads, which are conjugated with secondary antibody. Under optimum conditions, this assay is capable of detecting virus pictograms/well. A combination of nitrocellulose membrane with magnetic beads reduces analytical time and makes this assay stable and reproducible for point-of-care applications [97].

#### Thermometric biosensor

Thermometric biosensor measure the change in temperature after a biochemical reaction or in circulated medium. Earlier studies reported that the change in heat was monitored directly to measure the extent of reaction during catalysis or structural changes in molecules [98]. Thermometric biosensors use the capability of measuring temperature of thermometric devices, which led to the development of these sensors [99,100].

## Concluding remarks and future directions

Since post nine decades swine flu is responsible for many pandemics, several millions of deaths also create a major public concern into this area of research. It takes 2–3 days to show its full symptomatic condition and early stage detection becomes necessary for early treatment. Old methods are time consuming, mostly based on antigen–antibody titre, which makes them less sensitive and also have a problem of false negative results. Various kits have been developed for swine flu detection that is based on immunochromatographic techniques as shown in Table 1, which also shows average results but not as informational as provided by real-time PCR. Recently, a study reported the development of anti-HA Fab, a quenchbody (an immunosensor protein, which is fluorophore labelled antibody or segment of antibody shows a fluprescence response after quenching of dye from antibody as response of interaction between quenchbody and antigen) shows high fluorescence in the presence of HA antigen with the potential for sensor development [106]. A number of biosensors have been reported, which are promising for specific results with great sensitivity as reported in Table 2.

In future it would be better if some DNA-based sensor that can diagnose it in short time with great sensitivity are available in kit, as being provided for glucose biosensor and for many others also. Only then we can detect H1N1 early in effective manner to save a number of lives. Table 3

**Table 3:** Conventional methods for detection of A(H1N1) virus

Serial No	Detection method	Sample type for detection	Detection time	Gene/protein	Sensitivity/L.O.D	References
1	Cell culture	H1N1 Virus	7days	-	86-94%	[33]
2	ELISA	H1N1 Virus	4-5 hours	HA antibody	93.7%	[52]
3	RIDT kits	H1N1 Virus	< 30min	NA/antibody	1.13 HAU	[62]
4	RT-LAMP	Extracted RNA	3-4 hours	HA gene	93.8%	[70]
5	Real-Time PCR	Extracted RNA	3-4 hour	HA gene	5copies/reaction	[107]
6	HI assay	Sera	-	HA	92%	[110]
7	Conventional RT-PCR	Extracted RNA	3-4 hour	HA gene	1.0104 TCID50	[111]

RIDT= Rapid influenza detection test, H1=Hemagglutinin 1, RT-LAMP= Reverse transcription-Loop mediated isothermal amplification, HA= Hemagglutinin, NA= Neuraminidase, TCID= Tissue Culture Infective Dose

In summary, some points can be concluded as follow:

1. Real-time PCR is a standard test recommended by WHO but it takes time and reported L.O.D is 5 copies/reaction [107] and it varies with different probes. It is also expensive because reaction can be proceed in batches. RIDT is fast but its sensitivity is not good in comparison with others. It can differentiate between influenza A and B, but cannot differentiate between different subtypes of influenza A. Reported L.O.D is 103–104 PFU/ml and accuracy of results is <70% but some researchers also reported it as less sensitive for regular detection of influenza [108,109]. ELISA is better than HI assay but cannot detect viruses at early stage. Hemagglutinin inhibition assay and complment fixation methods show sensitivity and specificity of 91.2%, 38.7% and 25.7%, 85% respectively. Conventional RT-PCR is also a good method for detection but it also takes time. A list of all present detection methods for H1N1 is shown in Table 3 [110,111].
2. New methods are user friendly, cost effective and specific, which provides an easy point of care assay. Molecular combination with nanotechnology provides new diagnostic methods, which are very effective method for diagnosis. There are a number of biosensors specific for A (H1N1) pdm09 detection that are reported. Many sensors are based upon immunological properties (antigen–antibody) but DNA biosensors can be better choice if we make attempts as are reported for other infectious diseases [112]. All developed sensors are achieving higher sensitivity with time. Attempts should be made to develop it in a kit to detect in an easy, fast, sensitive, specific, time saving and cost-effective manner. Many places do not have facility of advance machines for the detection of swine flu but the development of easy detection kit can solve this problem.

## Abbreviations

FCA: feline calicivirus
HA: hemagglutunin
NA: neuraminidase
RGO: reduced graphene oxide
ssDNA: single-stranded DNA

## References

[1] Webster R.G., Peiris M., Chen H. and Guan Y. (2006) H5N1 outbreaks and enzootic influenza. Biodiversity 7, 51–55 10.1080/14888386.2006.9712795 PMC3291402 16494709

[2] Gamblin S.J. and Skehel J.J. (2010) Influenza hemagglutinin and neuraminidase membrane glycoproteins. J. Biol. Chem. 285, 28403–28409 10.1074/jbc.R110.129809 20538598 PMC2937864

[3] Bults M., Beaujean D.J., Kok G., Richardus J.H. and Voeten H.A. (2010) Mexican flu: risk perception in the general public, precautionary measures and trust in information provided by the government. Ned. Tijdschr. Geneeskd. 154, A1686 20482914

[4] Shope R.E. (1931) Swine influenza: III. Filtration experiments and etiology. J. Exp. Med. 54, 373–385 10.1084/jem.54.3.373 19869924 PMC2132000

[5] Garten R.J., Davis C.T., Russell C.A., Shu B., Lindstrom S., Balish A. et al. (2009) Antigenic and genetic characteristics of swine-origin 2009 A (H1N1) influenza viruses circulating in humans. Science. 325, 197–201 10.1126/science.1176225 19465683 PMC3250984

[6] Wise H.M., Hutchinson E.C., Jagger B.W., Stuart A.D., Kang Z.H., Robb N. et al. (2012) Identification of a novel splice variant form of the influenza A virus M2 ion channel with an antigenically distinct ectodomain. PLoS Pathog. 8, e1002998 10.1371/journal.ppat.1002998 23133386 PMC3486900

[7] Muramoto Y., Noda T., Kawakami E., Akkina R. and Kawaoka Y. (2013) Identification of novel influenza A virus proteins translated from PA mRNA. J. Virol. 87, 2455–2462 10.1128/JVI.02656-12 23236060 PMC3571384

[8] Bavagnoli L., Cucuzza S., Campanini G., Rovida F., Paolucci S., Baldanti F. et al. (2015) The novel influenza A virus protein PA-X and its naturally deleted variant show different enzymatic properties in comparison to the viral endonuclease PA. Nucleic Acids Res. 43, 9405–9417 10.1093/nar/gkv926 26384413 PMC4627086

[9] Memorandums M.L. (1980) A revision of the system of nomenclature for influenza viruses: a WHO memorandum. Bull. World Health Org. 58, 585–591 6969132 PMC2395936

[10] Ma W., Kahn R.E. and Richt J.A. (2009) The pig as a mixing vessel for influenza viruses: human and veterinary implications. J. Mole. Genetic Med.: An Int. J. Biomed. Res. 3, 158 PMC2702078 19565018

[11] Moreno A., Di Trani L., Faccini S., Vaccari G., Nigrelli D., Boniotti M.B. et al. (2011) Novel H1N2 swine influenza reassortant strain in pigs derived from the pandemic H1N1/2009 virus. Vet. Microbiol. 149, 472–477 10.1016/j.vetmic.2010.12.011 21208754

[12] Lim B.H. and Mahmood T.A. (2011) Influenza A H1N1 2009 (swine flu) and pregnancy. J. Obst. Gynecol. India 61, 386 10.1007/s13224-011-0055-2 22851818 PMC3295877

[13] Johnson N.P. and Mueller J. (2002) Updating the accounts: global mortality of the 1918-1920" Spanish" influenza pandemic. Bull. Hist. Med. 76, 105–115 10.1353/bhm.2002.0022 11875246

[14] Taubenberger J.K. and Morens D.M. (2006) 1918 Influenza: the mother of all pandemics. Revista Biomedica. 17, 69–79 10.3201/eid1201.050979 PMC3291398 16494711

[15] Pyle G.F. (1986) The diffusion of influenza: patterns and paradigms. Rowman Littlefield 12, 15–22

[16] Dunn F.L. (1958) Pandemic influenza in 1957: review of international spread of new Asian strain. J. Am. Med. Assoc. 166, 1140–1148 10.1001/jama.1958.02990100028006 13513331

[17] Viboud C., Simonsen L., Fuentes R., Flores J., Miller M.A. and Chowell G. (2016) Global mortality impact of the 1957–1959 influenza pandemic. J. Infect. Dis. 213, 738–745 10.1093/infdis/jiv534 26908781 PMC4747626

[18] Kilbourne E.D. (2006) Influenza pandemics of the 20th century. Emerg. Infect. Dis. 12, 9 10.3201/eid1201.051254 16494710 PMC3291411

[19] Reperant L.A., Moesker F.M. and Osterhaus A.D., Influenza: from zoonosis to pandemic. Eur. Respir. Soc. J. 2, 1–4 10.1183/23120541.00013-2016 PMC5005146 27730163

[20] Bults M., Beaujean D.J., Kok G., Richardus J.H. and Voeten H.A. (2010) Mexican flu: risk perception in the general public, precautionary measures and trust in information provided by the government. Ned. Tijdschr. Geneeskd. 154, A1686 20482914

[21] Guan Y., Vijaykrishna D., Bahl J., Zhu H., Wang J. and Smith G.J. (2010) The emergence of pandemic influenza viruses. Protein Cell 1, 9–13 21203993 10.1007/s13238-010-0008-z PMC4875113

[22] Seasonal Influenza (H1N1) – State/UT- wise, Year- wise number of cases and death from 2012 to 2019 (till 03rd March, 2019), https://ncdc.gov.in/showfile.php?lid=280. Accessed 3 March 2019

[23] Krammer F. and Palese P. (2015) Advances in the development of influenza virus vaccines. Nat. Rev. Drug Discov. 14, 167–182 10.1038/nrd4529 25722244

[24] Bose M.E., Beck E.T., Ledeboer N., Kehl S.C., Jurgens L.A., Patitucci T. et al. (2009) Rapid semiautomated subtyping of influenza virus species during the 2009 swine origin influenza A H1N1 virus epidemic in Milwaukee, Wisconsin. J. Clin. Microbiol. 47, 2779–2786 10.1128/JCM.00999-09 19641066 PMC2738075

[25] He J., Bose M.E., Beck E.T., Fan J., Tiwari S., Metallo J. et al. (2009) Rapid multiplex reverse transcription-PCR typing of influenza A and B virus, and subtyping of influenza A virus into H1, 2, 3, 5, 7, 9, N1 (human), N1 (animal), N2, and N7, including typing of novel swine origin influenza A (H1N1) virus, during the 2009 outbreak in Milwaukee, Wisconsin. J. Clin. Microbiol. 47, 2772–2778 10.1128/JCM.00998-09 19641063 PMC2738083

[26] MÈ Hamelin, M Baz, Abed Y., Couture C., Joubert P., É Beaulieu et al. (2010) Oseltamivir-resistant pandemic A/H1N1 virus is as virulent as its wild-type counterpart in mice and ferrets. PLoS Pathog. 6, e1001015 10.1371/journal.ppat.1001015 20661429 PMC2908621

[27] Furuse Y., Odagiri T., Okada T., Khandaker I., Shimabukuro K., Sawayama R. et al. (2010) Differentiation of human influenza A viruses including the pandemic subtype H1N1/2009 by conventional multiplex PCR. J. Virol. Methods 168, 94–97 10.1016/j.jviromet.2010.04.023 20447424

[28] Saylan Y., Erdem Ö, Ünal S. and Denizli A. (2019) An Alternative Medical Diagnosis Method: Biosensors for Virus Detection. Biosensors 9, 65 10.3390/bios9020065 PMC6627152 31117262

[29] Moulick A., Richtera L., Milosavljevic V., Cernei N., Haddad Y., Zitka O. et al. (2017) Advanced nanotechnologies in avian influenza: current status and future trends–a review. Anal. Chim. Acta 983, 42–53 10.1016/j.aca.2017.06.045 28811028 PMC7094654

[30] Sayhi M., Ouerghi O., Belgacem K., Arbi M., Tepeli Y., Ghram A. et al. (2018) Electrochemical detection of influenza virus H9N2 based on both immunomagnetic extraction and gold catalysis using an immobilization-free screen printed carbon microelectrode. Biosens. Bioelectron. 107, 170–177 10.1016/j.bios.2018.02.018 29455027

[31] Chauhan N., Narang J., Pundir S., Singh S. and Pundir C.S. (2013) Laboratory diagnosis of swine flu: a review. Artificial Cell Nanomed. Biotechnol. 41, 189–195 10.3109/10731199.2012.716063 23140089

[32] Miller E., Hoschler K., Hardelid P., Stanford E., Andrews N. and Zambon M. (2010) Incidence of 2009 pandemic influenza A H1N1 infection in England: a cross-sectional serological study. Lancet North Am. Ed. 375, 1100–1108 10.1016/S0140-6736(09)62126-7 20096450

[33] Roa P.L., Catalán P., Giannella M., de Viedma D.G., Sandonis V. and Bouza E. (2011) Comparison of real-time RT-PCR, shell vial culture, and conventional cell culture for the detection of the pandemic influenza A (H1N1) in hospitalized patients. Diagn. Microbiol. Infect. Dis. 69, 428–431 10.1016/j.diagmicrobio.2010.11.007 21396540

[34] Higgins A.D., Shaw C.J., Johnson J.G., Navarro A., Chapman N.A., Ewers S.D. et al. (2010) Monoclonal antibody kit for identification of the novel 2009 H1N1 influenza A virus. J. Clin. Microbiol. 48, 2677–2682 10.1128/JCM.00978-10 20519459 PMC2916606

[35] Bennett J.E., Dolin R. and Blaser M.J. (2014) In Mandell, Douglas, and Bennett’s Principles and Practice of Infectious Diseases: 2-Volume Set. Elsevier Health Sciences 2, 3904

[36] Miller E., Hoschler K., Hardelid P., Stanford E., Andrews N. and Zambon M. (2010) Incidence of 2009 pandemic influenza A H1N1 infection in England: a cross-sectional serological study. Lancet North Am. Ed. 375, 1100–1108 10.1016/S0140-6736(09)62126-7 20096450

[37] Mahony J.B. (2008) Detection of respiratory viruses by molecular methods. Clin. Microbiol. Rev. 21, 716–747 10.1128/CMR.00037-07 18854489 PMC2570148

[38] Janke B.H. (2000) Diagnosis of swine influenza. J. Swine Health Prod. 8, 79–84

[39] WORLD HEALTH ORGANIZATION. (1959) Expert Committee on Respiratory Virus Diseases. First Report, Stockholm, 11-15 August 1958. Expert Committee on Respiratory Virus Diseases. First Report, Stockholm, 11-15 August 1958

[40] Hoyle L. and Fairbrother B.W. (1937) Isolation of influenza virus and relation of antibodies to infection and immunity. Brit. Med. J. 1, 655 10.1136/bmj.1.3977.655 20780563 PMC2088494

[41] Morrison A.P., Shaw D.R., Kenney A.S. and Stokes J. Jr (1939) Complement-Fixation Studies on the Sera of Individuals Vaccinated with Active Virus of Human Influenza. Am. J. Med. Sci. 197, 253–260 10.1097/00000441-193902000-00014

[42] Eaton M.D. and Rickard E.R. (1941) Application of the complement-fixation test to the study of epidemic influenza. Am. J. Epidemiol. 33, 23–35 10.1093/oxfordjournals.aje.a118697

[43] Pyhälä R. and Kleemola M. (1976) The value of complement fixation and haemagglutination inhibition tests in the diagnosis of influenza A. Acta Virol. 20, 66–69 7943

[44] Fairbrother R.W. and Hoyle L. (1937) Observations on the Aetiology of Influenza. J. Pathol. Bacteriol. 44, 213–213 10.1002/path.1700440116

[45] Tulloch W.J. (1939) Observations on the Virus of Influenza, with a View to Elaborating a Simple Diagnostic Test Whereby Its Presence in the Respiratory Tract of Man May Be Revealed—Part I. Edinburgh Med. J. 46, 117–143 PMC5305391 29647653

[46] Dowdle W.R., Galphin J.C., Coleman M.T. and Schild G.C. (1974) A simple double immunodiffusion test for typing influenza viruses. Bull. World Health Organ. 51, 213 4218967 PMC2366274

[47] Schild G.C., Wood J.M. and Newman R.W. (1975) A single-radial-immunodiffusion technique for the assay of influenza haemagglutinin antigen: Proposals for an assay method for the haemagglutinin content of influenza vaccines. Bull. World Health Organ. 52, 223–231 816480 PMC2366365

[48] Williams M.S. (1993) Single-radial-immunodiffusion as an in vitro potency assay for human inactivated viral vaccines. Vet. Microbiol. 37, 253–262 10.1016/0378-1135(93)90027-5 8116186

[49] Choi Y., Lee S., Kwon S.Y., Lee Y., Park Y.K. and Ban S.J. (2017) Analysis of the proficiency of single radial immunodiffusion assays for quality control of influenza vaccines in Korea. Biologicals 50, 137–140 10.1016/j.biologicals.2017.08.001 29111376

[50] Rodda S.J., Gallichio H.A. and Hampson A.W. (1981) The single radial immunodiffusion assay highlights small antigenic differences among influenza virus hemagglutinins. J. Clin. Microbiol. 14, 479–482 10.1128/JCM.14.5.479-482.1981 6171580 PMC273972

[51] Lee B.W., Bey R.F., Baarsch M.J. and Simonson R.R. (1993) ELISA method for detection of influenza A infection in swine. J. Vet. Diagn. Invest. 5, 510–515 10.1177/104063879300500402 8286447

[52] Lu C.Y., Chang L.Y., Chen P.J., Xia N.S., Shao P.L. and Huang L.M. (2012) A highly specific ELISA for diagnosis of 2009 influenza A (H1N1) virus infections. J. Formos. Med. Assoc. 111, 693–697 10.1016/j.jfma.2011.11.029 23265748

[53] Rizzo F., Lovecchio C., Ingravalle F., Ceccarelli L., Sona B. and Mandola M.L. (2016) Swine influenza A serology: ELISA versus HI test. Int. J. Infect. Dis. 53, 105 10.1016/j.ijid.2016.11.263

[54] Ahmed S.R., Kim J., Suzuki T., Neethirajan S., Lee J. and Park E.Y. (2017) In situ self-assembly of gold nanoparticles on hydrophilic and hydrophobic substrates for influenza virus-sensing platform. Sci. Rep. 7, 44495 10.1038/srep44495 28290527 PMC5349514

[55] Julkunen I., Pyhälä R. and Hovi T. (1985) Enzyme immunoassay, complement fixation and hemagglutination inhibition tests in the diagnosis of influenza A and B virus infections. Purified hemagglutinin in subtype-specific diagnosis. J. Virol. Meth. 10, 75–84 10.1016/0166-0934(85)90091-6 3882733

[56] Pedersen J.C. (2014) Hemagglutination-inhibition assay for influenza virus subtype identification and the detection and quantitation of serum antibodies to influenza virus. Animal influenza virus, Humana Press, New York, NY, (pp. 11-25) 10.1007/978-1-4939-0758-8_2 24899416

[57] Reber A. and Katz J. (2013) Immunological assessment of influenza vaccines and immune correlates of protection. Expert Rev. Vaccines 12, 519–536 10.1586/erv.13.35 23659300 PMC9002926

[58] Zacour M., Ward B.J., Brewer A., Tang P., Boivin G., Li Y. et al. (2016) Standardization of hemagglutination inhibition assay for influenza serology allows for high reproducibility between laboratories. Clin. Vaccine Immunol. 23, 236–242 10.1128/CVI.00613-15 26818953 PMC4783428

[59] Muthana A.K. (2012) Comparison between Haemagglutination Inhibition and Complement Fixation Tests in Detecting Antibodies Responses Following Influenza Viral Infection. Egypt. Acad. J. Biol. Sci. 4, 35–38

[60] Julkunen I., Pyhälä R. and Hovi T. (1985) Enzyme immunoassay, complement fixation and hemagglutination inhibition tests in the diagnosis of influenza A and B virus infections. Purified hemagglutinin in subtype-specific diagnosis. J. Virol. Meth. 10, 75–84 10.1016/0166-0934(85)90091-6 3882733

[61] Prince H.E. and Leber A.L. (2003) Comparison of complement fixation and hemagglutination inhibition assays for detecting antibody responses following influenza virus vaccination. Clin. Diagn. Lab. Immunol. 10, 481–482 12738654 10.1128/CDLI.10.3.481-482.2003 PMC154979

[62] Lee G.C., Jeon E.S., Kim W.S., Le D.T., Yoo J.H. and Chong C.K. (2010) Evaluation of a rapid diagnostic test, NanoSign® Influenza A/B Antigen, for detection of the 2009 pandemic influenza A/H1N1 viruses. Virol. J. 7, 244 10.1186/1743-422X-7-244 20849665 PMC2949845

[63] Lucas P.M., Morgan O.W., Gibbons T.F., Guerrero A.C., Maupin G.M., Butler J.L. et al. (2011) Diagnosis of 2009 pandemic influenza A (pH1N1) and seasonal influenza using rapid influenza antigen tests, San Antonio, Texas, April–June 2009. Clin. Infect. Dis. 52, S116–S122 10.1093/cid/ciq027 21342882

[64] Baas C., Barr I.G., Fouchier R.A., Kelso A. and Hurt A.C. (2013) A comparison of rapid point-of-care tests for the detection of avian influenza A (H7N9) virus, 2013. Eurosurveillance 18, 20487 23725980

[65] Al Johani S.M., Al Balwi M., Al Alwan B., Al Hefdhi R. and Hajeer A. (2011) Validity of two rapid point of care influenza tests and direct fluorescence assay in comparison of real time PCR for swine of origin influenza virus. J. Infect. Public Health 4, 7–11 10.1016/j.jiph.2010.10.004 21338954

[66] Harmon K.M. and Yoon K.J. (1999) Application of PCR assay to differentiate two subtypes of swine influenza viruses. Swine Res. Rep. 42

[67] Poon L.L., Chan K.H., Smith G.J., Leung C.S., Guan Y., Yuen K.Y. et al. (2009) Molecular detection of a novel human influenza (H1N1) of pandemic potential by conventional and real-time quantitative RT-PCR assays. Clin. Chem. 55, 1555–1558 10.1373/clinchem.2009.130229 19439731 PMC7108475

[68] Harmon K., Bower L., Kim W.I., Pentella M. and Yoon K.J. (2010) A matrix gene–based multiplex real‐time RT‐PCR for detection and differentiation of 2009 pandemic H1N1 and other influenza A viruses in North America. Influenza Other Respir. Viruses 4, 405–410 10.1111/j.1750-2659.2010.00153.x 20958935 PMC4634613

[69] Notomi T., Okayama H., Masubuchi H., Yonekawa T., Watanabe K., Amino N. et al. (2000) Loop-mediated isothermal amplification of DNA. Nucleic Acids Res. 28, e63 10.1093/nar/28.12.e63 10871386 PMC102748

[70] Sharma V., Chaudhry D. and Kaushik S. (2018) Evaluation of clinical applicability of reverse transcription-loop-mediated isothermal amplification assay for detection and subtyping of Influenza A viruses. J. Virol. Methods 253, 18–25 10.1016/j.jviromet.2017.12.005 29253497 PMC7113880

[71] Wang J. (2008) Electrochemical glucose biosensors. Chem. Rev. 108, 814–825 10.1021/cr068123a 18154363

[72] Mehrotra P. (2016) Biosensors and their applications–A review. J. Oral Biol. Craniofacial Res. 6, 153–159 10.1016/j.jobcr.2015.12.002 PMC4862100 27195214

[73] Lee K.G., Lee T.J., Jeong S.W., Choi H.W., Heo N.S., Park J.Y. et al. (2012) Development of a plastic-based microfluidic immunosensor chip for detection of H1N1 influenza. Sensors 12, 10810–10819 10.3390/s120810810 23112630 PMC3472858

[74] Critchley P. and Dimmock N.J. (2004) Binding of an influenza A virus to a neomembrane measured by surface plasmon resonance. Bioorg. Med. Chem. 12, 2773–2780 10.1016/j.bmc.2004.02.042 15110858

[75] Su L.C., Chang C.M., Tseng Y.L., Chang Y.F., Li Y.C., Chang Y.S. et al. (2012) Rapid and highly sensitive method for influenza A (H1N1) virus detection. Anal. Chem. 84, 3914–3920 10.1021/ac3002947 22401570

[76] Bai H., Wang R., Hargis B., Lu H. and Li Y. (2012) A SPR aptasensor for detection of avian influenza virus H5N1. Sensors 12, 12506–12518 10.3390/s120912506 23112728 PMC3478855

[77] Pang Y., Rong Z., Wang J., Xiao R. and Wang S. (2015) A fluorescent aptasensor for H5N1 influenza virus detection based-on the core–shell nanoparticles metal-enhanced fluorescence (MEF). Biosens. Bioelectron. 66, 527–532 10.1016/j.bios.2014.10.052 25506900

[78] Shi L., Sun Q., He J.A., Xu H., Liu C., Zhao C. et al. (2015) Development of SPR biosensor for simultaneous detection of multiplex respiratory viruses. Biomed. Mater. Eng. 26, S2207–S2216 26406000 10.3233/BME-151526

[79] Nidzworski D., Pranszke P., Grudniewska M., Król E. and Gromadzka B. (2014) Universal biosensor for detection of influenza virus. Biosens. Bioelectron. 59, 239–242 10.1016/j.bios.2014.03.050 24732601

[80] Hai W., Goda T., Takeuchi H., Yamaoka S., Horiguchi Y., Matsumoto A. et al. (2017) Specific recognition of human influenza virus with PEDOT bearing sialic acid-terminated trisaccharides. ACS Appl. Mater. Interfaces 9, 14162–14170 10.1021/acsami.7b02523 28379685

[81] Bonanni A., Pividori M.I. and Del Valle M. (2010) Impedimetric detection of influenza A (H1N1) DNA sequence using carbon nanotubes platform and gold nanoparticles amplification. Analyst 135, 1765–1772 10.1039/c000532k 20458407

[82] Singh R., Hong S. and Jang J. (2017) Label-free detection of influenza viruses using a reduced graphene oxide-based electrochemical immunosensor integrated with a microfluidic platform. Sci. Rep. 7, 42771 10.1038/srep42771 28198459 PMC5309888

[83] Nidzworski D., Siuzdak K., Niedziałkowski P., Bogdanowicz R., Sobaszek M., Ryl J. et al. (2017) A rapid-response ultrasensitive biosensor for influenza virus detection using antibody modified boron-doped diamond. Sci. Rep. 7, 15707 10.1038/s41598-017-15806-7 29146948 PMC5691202

[84] Lee W., Kang T., Kim S.H. and Jeong J. (2018) An Antibody-Immobilized Silica Inverse Opal Nanostructure for Label-Free Optical Biosensors. Sensors 18, 307 10.3390/s18010307 PMC5796272 29361683

[85] Pohanka M. and Skládal P. (2008) Electrochemical biosensors–principles and applications. J. Appl. Biomed. (De Gruyter Open) 6, 57–64 10.32725/jab.2008.008

[86] Singh S., Kaushal A., Khare S., Kumar P. and Kumar A. (2014) Gold–mercaptopropionic acid–polyethylenimine composite based DNA sensor for early detection of rheumatic heart disease. Analyst 139, 3600–3606 10.1039/C4AN00324A 24875529

[87] Dash S.K., Sharma M., Khare S. and Kumar A. (2013) Omp85 genosensor for detection of human brain bacterial meningitis. Biotechnol. Lett. 35, 929–935 10.1007/s10529-013-1161-2 23471585

[88] Lee D., Chander Y., Goyal S.M. and Cui T. (2011) Carbon nanotube electric immunoassay for the detection of swine influenza virus H1N1. Biosens. Bioelectron. 26, 3482–3487 10.1016/j.bios.2011.01.029 21354779 PMC7126489

[89] Bai C., Lu Z., Jiang H., Yang Z., Liu X., Ding H. et al. (2018) Aptamer selection and application in multivalent binding-based electrical impedance detection of inactivated H1N1 virus. Biosens. Bioelectron. 110, 162–167 10.1016/j.bios.2018.03.047 29609164

[90] Mikuła E., Silva C.E., Kopera E., Zdanowski K., Radecki J. and Radecka H. (2018) Highly sensitive electrochemical biosensor based on redox-active monolayer for detection of anti-hemagglutinin antibodies against swine-origin influenza virus H1N1 in sera of vaccinated mice. BMC Vet. Res. 14, 328 10.1186/s12917-018-1668-9 30400888 PMC6218974

[91] Metaferia B., Wei J.S., Song Y.K., Evangelista J., Aschenbach K., Johansson P. et al. (2013) Development of peptide nucleic acid probes for detection of the HER2 oncogene. PLoS One 8, e58870 10.1371/journal.pone.0058870 23593123 PMC3622650

[92] García-Martinez G., Bustabad E.A., Perrot H., Gabrielli C., Bucur B., Lazerges M. et al. (2011) Development of a mass sensitive quartz crystal microbalance (QCM)-based DNA biosensor using a 50 MHz electronic oscillator circuit. Sensors 11, 7656–7664 10.3390/s110807656 22164037 PMC3231718

[93] Pohanka M. (2017) The Piezoelectric Biosensors: Principles and Applications. Int. J. Electrochem. Sci. 12, 496–506 10.20964/2017.01.44

[94] Pohanka M. (2018) Overview of piezoelectric biosensors, immunosensors and DNA sensors and their applications. Materials 11, 448 10.3390/ma11030448 PMC5873027 29562700

[95] Hewa T.M., Tannock G.A., Mainwaring D.E., Harrison S. and Fecondo J.V. (2009) The detection of influenza A and B viruses in clinical specimens using a quartz crystal microbalance. J. Virol. Methods 162, 14–21 10.1016/j.jviromet.2009.07.001 19628008 PMC7112868

[96] Erofeev A.S., Gorelkin P.V., Kolesov D.V., Kiselev G.A., Dubrovin E.V. and Yaminsky I.V. (2019) Label-free sensitive detection of influenza virus using PZT discs with a synthetic sialylglycopolymer receptor layer. Royal Soc. Open Sci. 6, 190255 10.1098/rsos.190255 31598281 PMC6774986

[97] Hong H.B., Krause H.J., Song K.B., Choi C.J., Chung M.A., Son S.W. et al. (2011) Detection of two different influenza A viruses using a nitrocellulose membrane and a magnetic biosensor. J. Immunol. Methods 365, 95–100 10.1016/j.jim.2010.12.005 21182841

[98] Grime J.K. (1985) Thermodynamics, thermochemistry and calorimetry. In Analytical solution calorimetry(Grime J.K., ed.), pp. 1–29, Wiley, New York, New York

[99] Mosbach K. and Danielsson B. (1974) An enzyme thermistor. Biochim. Biophys. Acta Enzymol. 364, 140–145 10.1016/0005-2744(74)90141-7 4373068

[100] Ramanathan K. and Danielsson B. (2001) Principles and applications of thermal biosensors. Biosens. Bioelectron. 16, 417–423 10.1016/S0956-5663(01)00124-5 11672656

[101] Miyoshi-Akiyama T., Narahara K., Mori S., Kitajima H., Kase T., Morikawa S. et al. (2010) Development of an immunochromatographic assay specifically detecting pandemic H1N1 (2009) influenza virus. J. Clin. Microbiol. 48, 703–708 10.1128/JCM.02262-09 20071549 PMC2832444

[102] Choi Y.J., Kim H.J., Park J.S., Oh M.H., Nam H.S., Kim Y.B. et al. (2010) Evaluation of new rapid antigen test for detection of pandemic influenza A/H1N1 2009 virus. J. Clin. Microbiol. 48, 2260–2262 10.1128/JCM.02392-09 20357213 PMC2884507

[103] Patel P., Graser E., Robst S., Hillert R., Meye A., Hillebrand T. et al. (2011) rapidSTRIPE H1N1 test for detection of the pandemic swine origin influenza A (H1N1) virus. J. Clin. Microbiol. 49, 1591–1593 10.1128/JCM.02563-10 21248098 PMC3122832

[104] Mizuike R., Sasaki T., Baba K., Iwamoto H., Shibai Y., Kosaka M. et al. (2011) Development of two types of rapid diagnostic test kits to detect the hemagglutinin or nucleoprotein of the swine-origin pandemic influenza A virus H1N1. Clin. Vaccine Immunol. 18, 494–499 10.1128/CVI.00269-10 21228147 PMC3067372

[105] Yu S.T., Bui C.T., Nguyen A.V., Trinh T.T. and Yeo S.J. (2018) Clinical evaluation of rapid fluorescent diagnostic immunochromatographic test for influenza A virus (H1N1). Sci. Rep. 8, 13468 10.1038/s41598-018-31786-8 30194330 PMC6128899

[106] Jeong H.J., Dong J. and Ueda H. (2019) Single-Step Detection of the Influenza Virus Hemagglutinin Using Bacterially-Produced Quenchbodies. Sensors 19, 52 10.3390/s19010052 PMC6338965 30583603

[107] Shu B., Wu K.H., Emery S., Villanueva J., Johnson R., Guthrie E. et al. (2011) Design and performance of the CDC real-time reverse transcriptase PCR swine flu panel for detection of 2009 A (H1N1) pandemic influenza virus. J. Clin. Microbiol. 49, 2614–2619 10.1128/JCM.02636-10 21593260 PMC3147828

[108] Kwon D., Shin K., Kwon M., Oh H.B., Kang C. and Lee J.Y. (2011) Development and evaluation of a rapid influenza diagnostic test for the pandemic (H1N1) 2009 influenza virus. J. Clin. Microbiol. 49, 437–438 10.1128/JCM.01628-10 20980580 PMC3020471

[109] Kuo C.Y., Huang Y.C., Huang C.G., Tsao K.C. and Lin T.Y. (2011) Symptomatic predictors for 2009 influenza A virus (H1N1) infection with an emphasis for patients with a negative rapid diagnostic test. PLoS One 6, e28102 10.1371/journal.pone.0028102 22164233 PMC3229543

[110] Veguilla V., Hancock K., Schiffer J., Gargiullo P., Lu X., Aranio D. et al. (2011) Sensitivity and specificity of serologic assays for detection of human infection with 2009 pandemic H1N1 virus in US populations. J. Clin. Microbiol. 49, 2210–2215 10.1128/JCM.00229-11 21471339 PMC3122722

[111] Poon L.L., Chan K.H., Smith G.J., Leung C.S., Guan Y., Yuen K.Y. et al. (2009) Molecular detection of a novel human influenza (H1N1) of pandemic potential by conventional and real-time quantitative RT-PCR assays. Clin. Chem. 55, 1555–1558 10.1373/clinchem.2009.130229 19439731 PMC7108475

[112] Hideshima S., Hinou H., Ebihara D., Sato R., Kuroiwa S., Nakanishi T. et al. (2013) Attomolar detection of influenza A virus hemagglutinin human H1 and avian H5 using glycan-blotted field effect transistor biosensor. Anal. Chem. 85, 5641–5644 10.1021/ac401085c 23675869

